# Effects of KCa channels on biological behavior of trophoblasts

**DOI:** 10.1515/biol-2022-0462

**Published:** 2022-09-03

**Authors:** Xiaolei Zhang, Meitao Yang, Dan Lv, Yin Xie, Yanan Sun, Yanling Zhang, Mengzhou He, Haiyi Liu, Fanfan Li, Dongrui Deng

**Affiliations:** Department of Gynecology and Obstetrics, Tongji Hospital, Tongji Medical College, Huazhong University of Science and Technology, No. 1095, Jiefang Ave, Wuhan, Hubei, China; Department of Gynecology and Obstetrics, Zhongnan Hospital, Wuhan University, Wuhan, 430071, China

**Keywords:** trophoblast, chorionic villi, BKCa channels, IKCa channels, SKCa channels

## Abstract

The Ca^2+^-activated potassium (KCa) channels are involved in many cellular functions, but their roles in trophoblasts are unclear. This study aimed to clarify the effects of KCa channels on the biological behavior of trophoblasts. The localization and expression of the three types of KCa channels, including large-conductance KCa channels (BKCa), intermediate-conductance KCa channels (IKCa), and small-conductance KCa channels (SKCa), were detected in human chorionic villi taken from pregnant women between 5 and 8 weeks of gestation (*n* = 15) and HTR-8/SVneo cells. The effects of KCa channels on proliferation, apoptosis, and migration of HTR-8/SVneo cells were examined by using the activators or inhibitors of KCa channels. Results showed that KCa channels were mainly localized on the membrane and in the cytoplasm of trophoblasts in human chorionic villi and HTR-8/SVneo cells. The proliferation and migration of HTR-8/SVneo cells were inhibited by activating KCa channels. Apoptosis of trophoblasts was promoted through activating BKCa channels but was not affected by neither activating nor inhibiting IKCa and SKCa channels. This study substantiated the abovementioned biological roles of KCa channels in trophoblast cells, which is fundamental to further research on whether dysfunction of KCa channels is involved in the pathogenesis of pregnancy-related complications.

## 1 Introduction

Trophoblast cells are the major building blocks of the developing placenta, which is a transient organ that plays a pivotal role in fetal growth during pregnancy. There are three major trophoblast subpopulations: cytotrophoblast (CTB), syncytiotrophoblast (STB), and extravillous cytotrophoblast (EVT) [1]. During trophoblast differentiation, CTB functions as a stem cell-like progenitor cell. In floating villi, CTB fuses to form STB, which serves as the main barrier against pathogens and participates in the exchange of the gas and nutrient between mother and fetus; in anchoring villi, it undergoes a modified form of epithelial-to-mesenchymal transition into EVT [2]. EVTs participate in uterine spiral artery remodeling [3,5]. In this process, EVTs invade the spiral arteries during early gestation and induce fibrinoid necrosis of the vessel walls. Eventually, these spiral arteries lose continuous endothelial lining and muscular and elastic tissue and often contain mural thrombi [3,4]. Dysfunction of trophoblast cells underlies placenta-based pregnancy complications, including recurrent miscarriage, preterm birth, preeclampsia (PE), and fetal growth restriction (FGR) [5,6]. However, molecular mechanisms featured in trophoblast behavior include proliferation, differentiation, apoptosis, invasion, and migration, remain elusive [7].

The Ca^2+^-activated potassium channels (KCa), which are activated by the increased intracellular concentration of free calcium, are broadly classified into three categories: large-conductance KCa channels (BKCa), intermediate-conductance KCa channels (IKCa), and small-conductance KCa channels (SKCa) [8,9]. Dysfunction of KCa channels has been widely reported to be associated with neurological and cardiovascular diseases, as well as the onset of PE [10–13]. Steroid hormone-mediated upregulation of KCa channels was negatively regulated by hypoxia-induced reactive oxygen species (ROS) production. However, this effect was reversed by ROS inhibitors, suggesting that abnormal expression of KCa channels might be associated with maladaptation of uterine vascular hemodynamics increased under the hypoxic circumstance of PE [10]. Hu et al. disclosed that chronic hypoxia during gestation upregulated DNA methyltransferase expression and activity, resulting in functional repression of the KCNMB1 gene and BKCa channels, which ultimately led to maladjustment of uterine arteries [11]. Our research team found that BKCa, IKCa, and SKCa channels were localized on both endothelium and smooth muscles of placental chorionic plate arteries; expression of these three channels was downregulated in PE placenta compared to that of the healthy control group [12,14].

Recent studies suggested that KCa channels could act as novel regulators of specific pathways involved in proliferation, differentiation, apoptosis, and invasion of various cancer cells [15]. As the biological behavior of EVTs shares many similar characteristics with cancer cells [6,16], it is reasonable to speculate that the well-functioning of KCa channels may be fundamental for trophoblasts. Dysfunction of trophoblast cells leads to defective spiral artery remodeling, which underlies the pathogenesis of PE and FGR [16]. Identifying the expression and functional roles of KCa channels in trophoblast cells might aid uncovering the pathogenesis of placenta-based pregnancy complications.

In this study, we aimed to explore whether KCa channels are expressed in human chorionic villi in early pregnancy and their effects on the biological activities of EVTs.

## 2 Materials and methods

### 2.1 Procurement of chorionic villi

Chorionic villus samples were collected from healthy pregnant women (*n* = 15) who requested voluntary pregnancy termination at 5–8 weeks of gestation in Tongji Hospital, Wuhan, China. Samples were collected immediately after induced abortion and placed into 4% paraformaldehyde (Servicebio, China) for immunohistochemical analysis.


**Informed consent:** Informed consent has been obtained from all individuals included in this study.
**Ethical approval:** The research related to human use has been complied with all the relevant national regulations, institutional policies and in accordance with the tenets of the Helsinki Declaration, and has been approved by the authors’ institutional review board or equivalent committee (IRB ID: TJ-C20180201).

### 2.2 Immunohistochemistry

Samples were fixed in 4% paraformaldehyde and embedded in paraffin. Serial sections were then cut and deparaffinized. Immunohistochemistry was performed using SP (streptavidin–peroxidase–biotin) staining. Sections were blocked with 5% bovine serum albumin (BSA, Gibco, USA) for 30 min at 37°C and incubated overnight with the primary antibodies. The primary antibodies included rabbit anti-BKCa α (APC-107, 1:100), BKCa β1 (APC-036, 1:100), IKCa (APC-064, 1:100), and SKCa (APC-025, 1:100), all of which were provided by Alomone Labs Company in Israel. They were subsequently incubated with appropriate secondary antibodies against Rabbit IgG at 37°C for 30 min. Sections were incubated with isotype IgG, and the same secondary antibody served as negative controls. The expression of target proteins was detected by a peroxide-conjugated streptavidin system with 3,3-diaminobenzidine as substrate (Zhongshan Goldenbridge Biotechnology, China). The presence of brownish-yellow granules in the cytoplasm or nucleus was considered positive.

### 2.3 Cell culture

HTR-8/SVneo cells (Cell Collection Center of Wuhan University, China), the human EVT trophoblast cell line, were cultured in Dulbecco’s modified eagle medium (DMEM)/High Glucose medium (Hyclone, USA) supplemented with 10% fetal bovine serum (FBS; Gibco, USA), 50 U/mL penicillin, and 50 μg/mL streptomycin (Sigma-Aldrich, St. Louis, MO, USA) at 37°C in a 5% CO_2_ incubator. Cells between 24 and 30 passages were used in this study [17].

### 2.4 Immunofluorescence

HTR-8/SVneo cells were seeded on coverslips and left to acclimate overnight. Then, cells were permeabilized with 0.5% TritonX-100 for 5 min before being blocked with 5% BSA for 1 h. The coverslips were incubated with primary antibody against cytokeratin 7 (CK7, 1:50; Abcam, UK) and anti-vimentin (1:50; Abcam), which were used for the confirmation of EVTs, as well as with other primary antibodies against BKCa α (1:50; Alomone Labs), BKCa β1 (1:50; Alomone Labs), IKCa (1:50; Alomone Labs), and SKCa (1:50; Alomone Labs) at 4°C overnight. After washing with phosphate-buffered saline (PBS) three times, the coverslips were incubated with fluorescence-conjugated secondary antibody (Servicebio) at room temperature for 1 h and stained with 4′,6-diamidino-2-phenylindole (DAPI; Servicebio) for 10 min. The coverslips were observed and imaged under a fluorescent microscope (Olympus IX73, Tokyo, Japan). The same procedure was repeated three times, independently.

### 2.5 Cell counting kit-8 (CCK-8) assay

For cell proliferation assessment, CCK-8 assay was conducted in the presence of NS1619 (BKCa channel activator), NS309 (IKCa channel and SKCa channel activator), ibTX (BKCa channel inhibitor), TRAM34 (IKCa channel inhibitor), and Apamin (SKCa channel inhibitor) [8,9,18]. First, 5 × 10^3^ HTR-8/SVneo cells per well were seeded into 96-well plates and maintained in DMEM/High Glucose medium supplemented with 10% FBS at 37°C in a 5% CO_2_ incubator overnight to acclimate. Then, HTR-8/SVneo cells were incubated in a complete medium containing 100 μM NS1619 (ab141824; Abcam), 100 μM NS309 (ab120371; Abcam), 3 nM ibTX (ab120379; Abcam), 25 μM TRAM34 (ab141885; Abcam), and 2.5 μM Apamin (ab120268; Abcam) at 37°C with 5% CO_2_ for 12, 24, and 48 h, respectively. All reagents were freshly prepared from stock solutions on the day of the experiment and dissolved in dimethyl sulfoxide (DMSO). The control group was added with the same volume of DMSO. Finally, 10 μL of CCK-8 reagent (Dojido Laboratories, Japan) was added to each well 2 h before the target detection time, and the absorbance value (OD) was measured at 450 nm after 2 h of incubation. OD value was determined on a Microplate Reader (Thermo Labsystems, USA). All experiments were done in triplicate wells at least thrice, independently.

### 2.6 Flow cytometry analysis

HTR-8/SVneo cells were incubated in a complete medium containing 100 μM NS1619, 100 μM NS309, 3 nM ibTX, 25 μM TRAM34, and 2.5 μM Apamin at 37°C with 5% CO_2_ for 12, 24, and 48 h, respectively. Apoptosis was detected by Annexin V-FITC Apoptosis Detection Kit (Becton Dickinson, USA). Briefly, HTR-8/SVneo cells were collected and washed twice with PBS. Then, 200 μL of binding buffer suspension was added to the treated cells. After that, 5 μL of Annexin V-FITC and 5 μL of propidium iodide were added to each group, and cultures were incubated at 37°C for 10 min in the dark. Afterward, cell apoptosis rate was monitored with a flow cytometer (Becton Dickinson, USA), and flowJo software was used for flow cytometry analysis. All experiments were repeated thrice, independently.

### 2.7 Transwell assay

The migration ability of HTR-8/SVneo cells was estimated with 8 μm pore-sized membranes (Corning, USA) using the transwell chamber. Briefly, HTR-8/SVneo cells were resuspended in the serum-free medium that contained 100 μM NS1619, 100 μM NS309, 3 nM ibTX, 25 μM TRAM34, 2.5 μM Apamin or DMSO of the same volume (100 μL/well) in the upper chamber, respectively. Simultaneously, the lower chamber was placed into a 500 μL complete culture medium. After 24 h of incubation at 37°C in a 5% CO_2_ incubator, HTR-8/SVneo cells that remained on the top side of the upper chamber were wiped off using a wet cotton-tipped swab. Then, HTR-8/SVneo cells that had migrated to the bottom side of the upper chamber were fixed with 4% paraformaldehyde and dyed with 0.5% crystal violet. The migrating HTR-8/SVneo cells were photographed under an inverted microscope (Olympus). At least three fields were randomly selected for cell counting per chamber. All experiments were repeated thrice independently.

### 2.8 Data analysis

Data were analyzed using SPSS 18.0 software (IBM, Armonk, NY, USA). All results were expressed as mean ± standard deviation (SD) of at least three independent experiments. Independent samples’ *t*-test was adopted for between-group comparison; one-way analysis of variance was used for multi-group comparison, and Fisher’s least significant difference *t*-test was used for post hoc pairwise comparison. *P* < 0.05 was considered statistically significant.

## 3 Results

### 3.1 Location of KCa channels in human villus tissues

**Figure 1:**
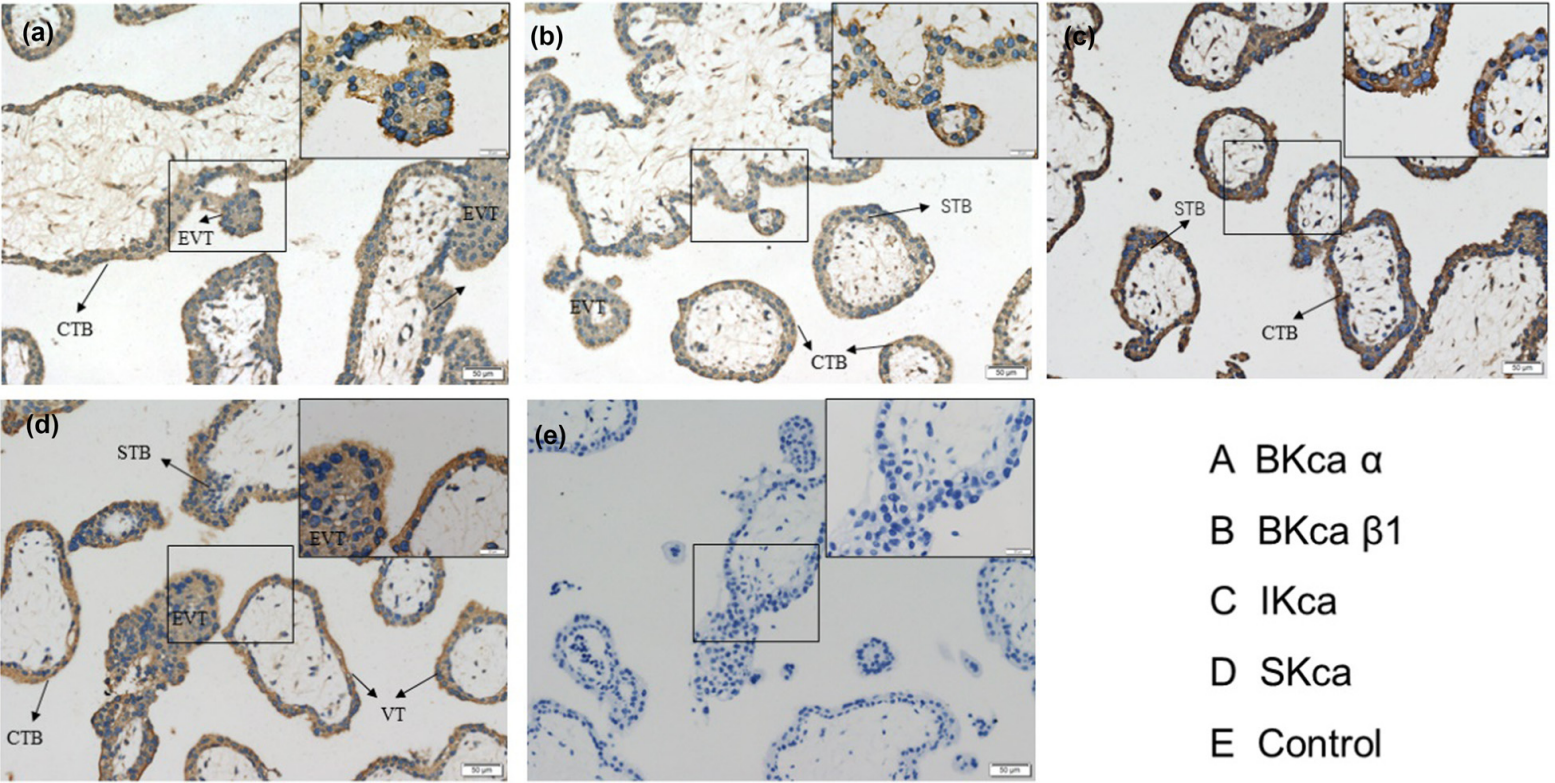
Expression of KCa channels in human villus tissues. Immunohistochemical detection of different types of KCa channels (BKCa α, BKCa β1, IKCa, and SKCa) and the negative control in human villus tissues.

Immunohistochemistry staining showed that BKCa α, BKCa β1, IKCa, and SKCa channels were expressed in CTBs, STBs, and EVTs, especially in EVTs. The negative result was observed in the control (Figure 1). Therefore, the human EVT cell line HTR-8/SVneo was selected for the sequent experiments.

### 3.2 Expression of KCa channels in HTR-8/SVneo cells

**Figure 2:**
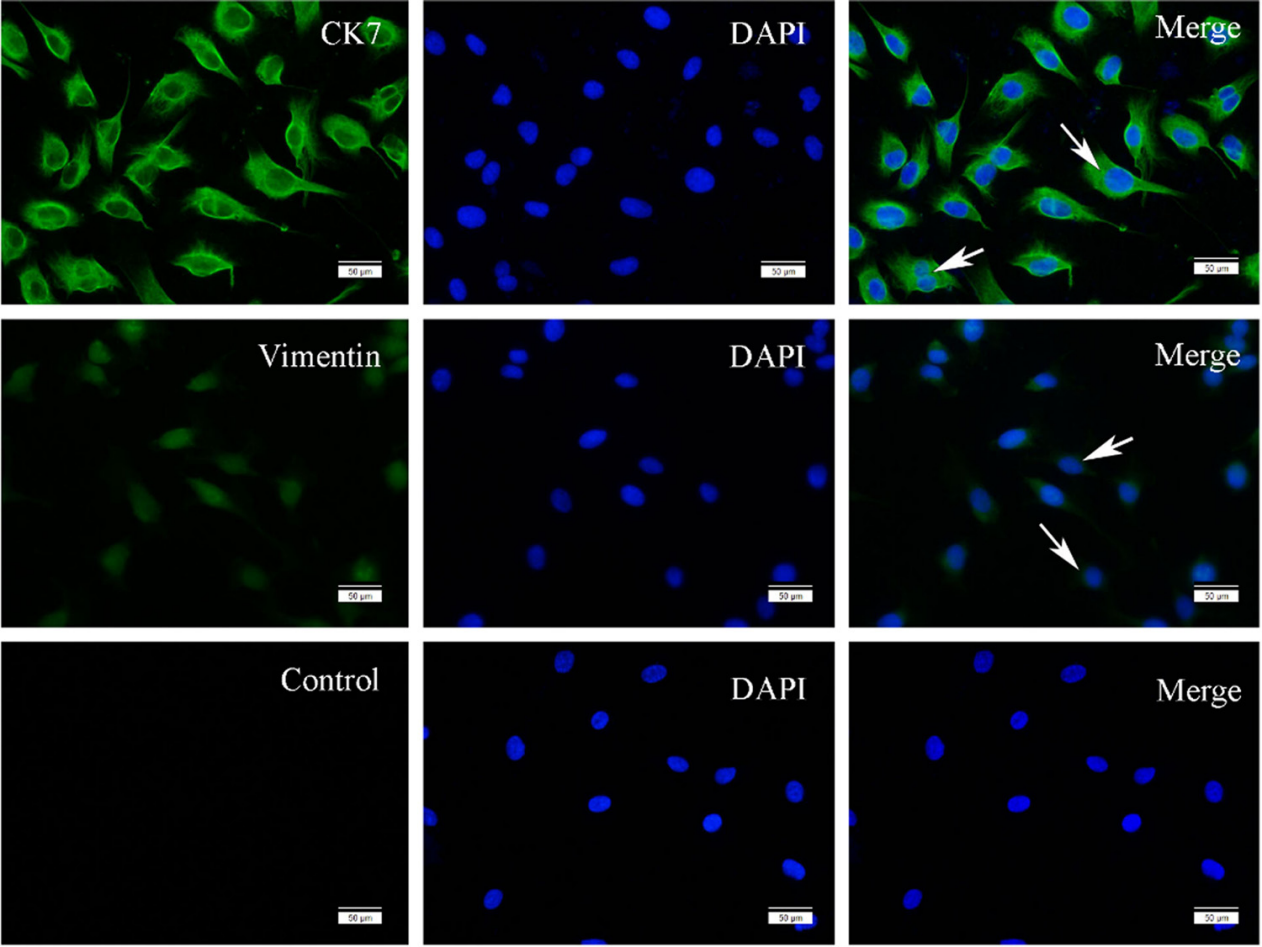
Localization and expression of CK7 and vimentin in HTR-8/SVneo cell line. CK7 and vimentin localization were detected by immunostaining with anti-CK 7 and anti-vimentin (green) under the fluorescence microscope, respectively. Cells were stained with DAPI to visualize nuclei (blue). Arrow indicates the positive staining.

**Figure 3:**
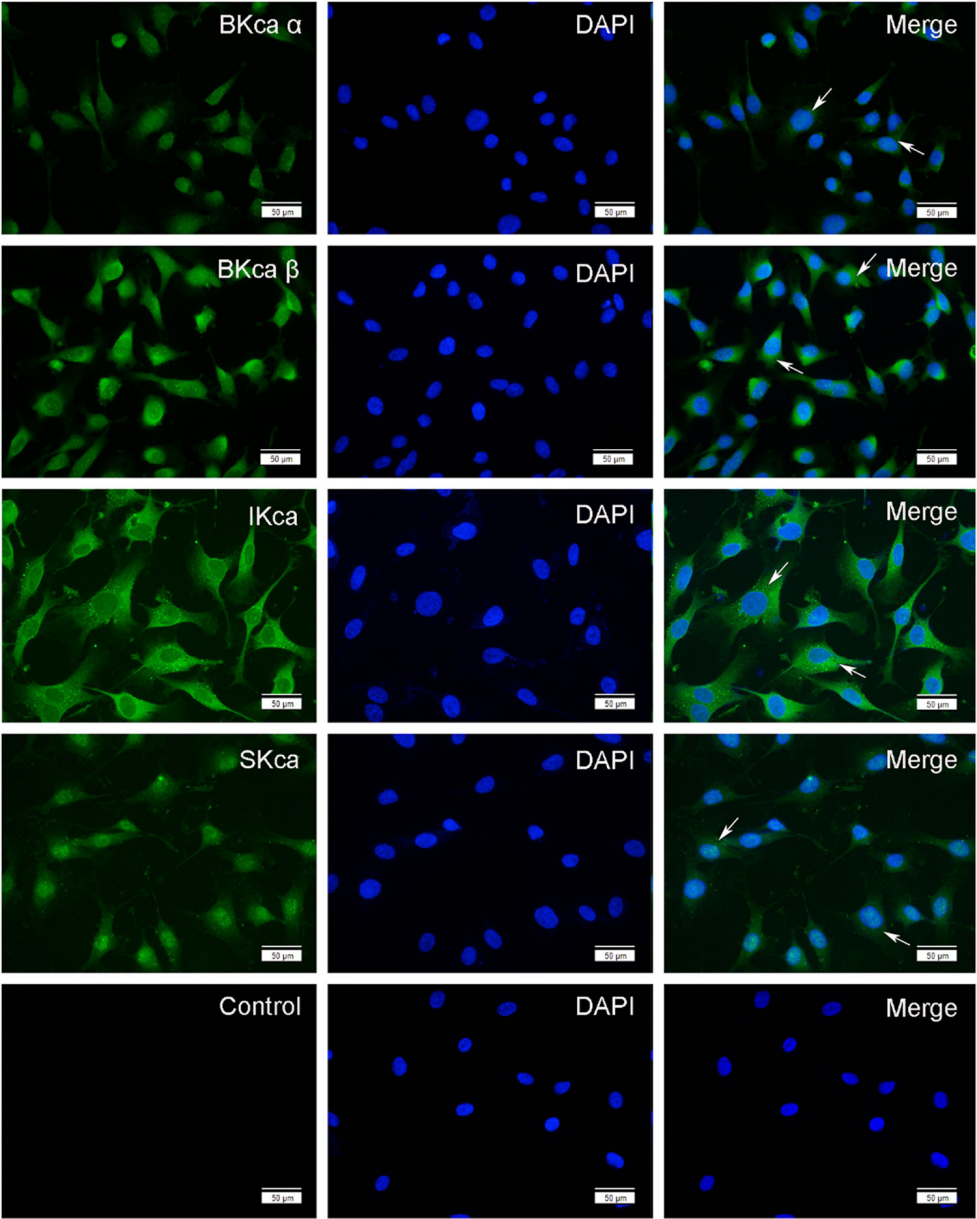
Localization and expression of KCa channels in HTR-8/SVneo cells. BKCa α and β1 subunits, IKCa channels, SKCa channels, and the control in HTR-8/SVneo cells were observed under the fluorescence microscope. Cells were stained with DAPI to visualize nucleus (blue) and immunolabeled with Anti-KCa antibodies (green), respectively. Arrow indicates the positive staining.

Immunofluorescence was performed to confirm the expression of the markers of EVTs-CK7 and vimentin as well as KCa subunits in HTR-8/SVneo cells. Results showed that the selected cell line was indeed the HTR-8/SVneo cell with very high purity (Figure 2). BKCa α subunits were less expressed on the membrane or in the cytoplasm of HTR-8/SVneo cells compared with BKCa β1 subunit. IKCa and SKCa channels were mainly distributed on the cell membrane but less expressed in the cytoplasm (Figure 3).

### 3.3 Effect of KCa channels on the proliferation of HTR-8/SVneo cells

**Figure 4:**
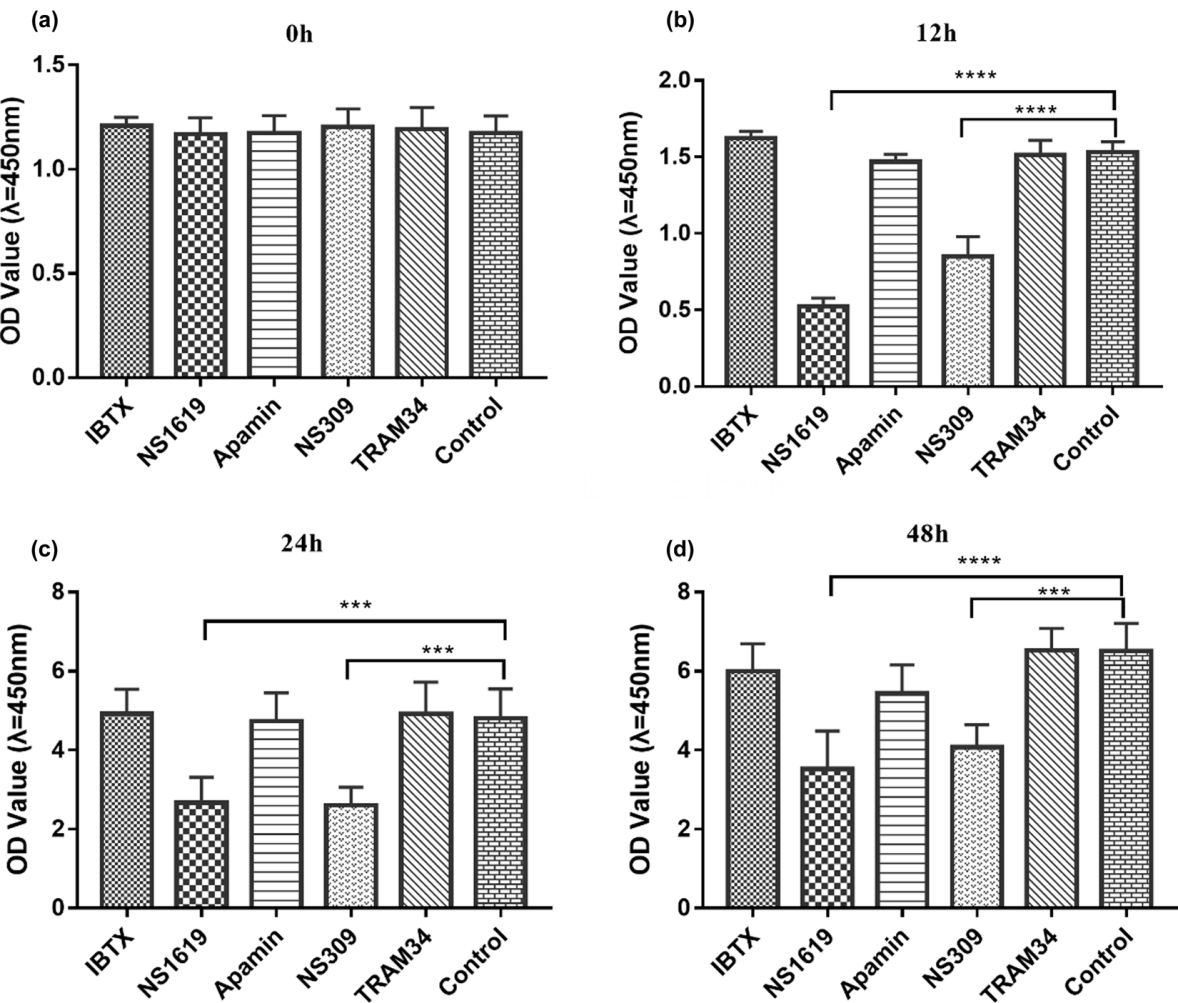
CCK-8 kits to evaluate effects of KCa channel activators and inhibitors on proliferation of HTR-8/SVneo cells. OD values were measured at 450 nm after HTR-8/SVneo cells had been treated with NS1619, NS309, ibTX, TRAM34, and Apamin for 0 h (a), 12 h (b), 24 h (c), and 48 h (d), respectively. Data are displayed as mean ± SD. ***P < 0.001, ****P < 0.0001. All experiments were done in triplicate wells for at least three times, independently.

After incubation with NS1619 or NS309 for 12, 24, and 48 h, the cell proliferation was significantly inhibited (*P* < 0.001). It suggested that activating BKCa, IKCa, and SKCa channels suppressed the proliferation of HTR-8/SVneo cells. However, no significant difference was observed in the proliferation of HTR-8/SVneo cells after inhibiting BKCa, IKCa, or SKCa channels (Figure 4).

### 3.4 Apoptosis of HTR-8/SVneo cells after intervention on KCa channels

**Figure 5:**
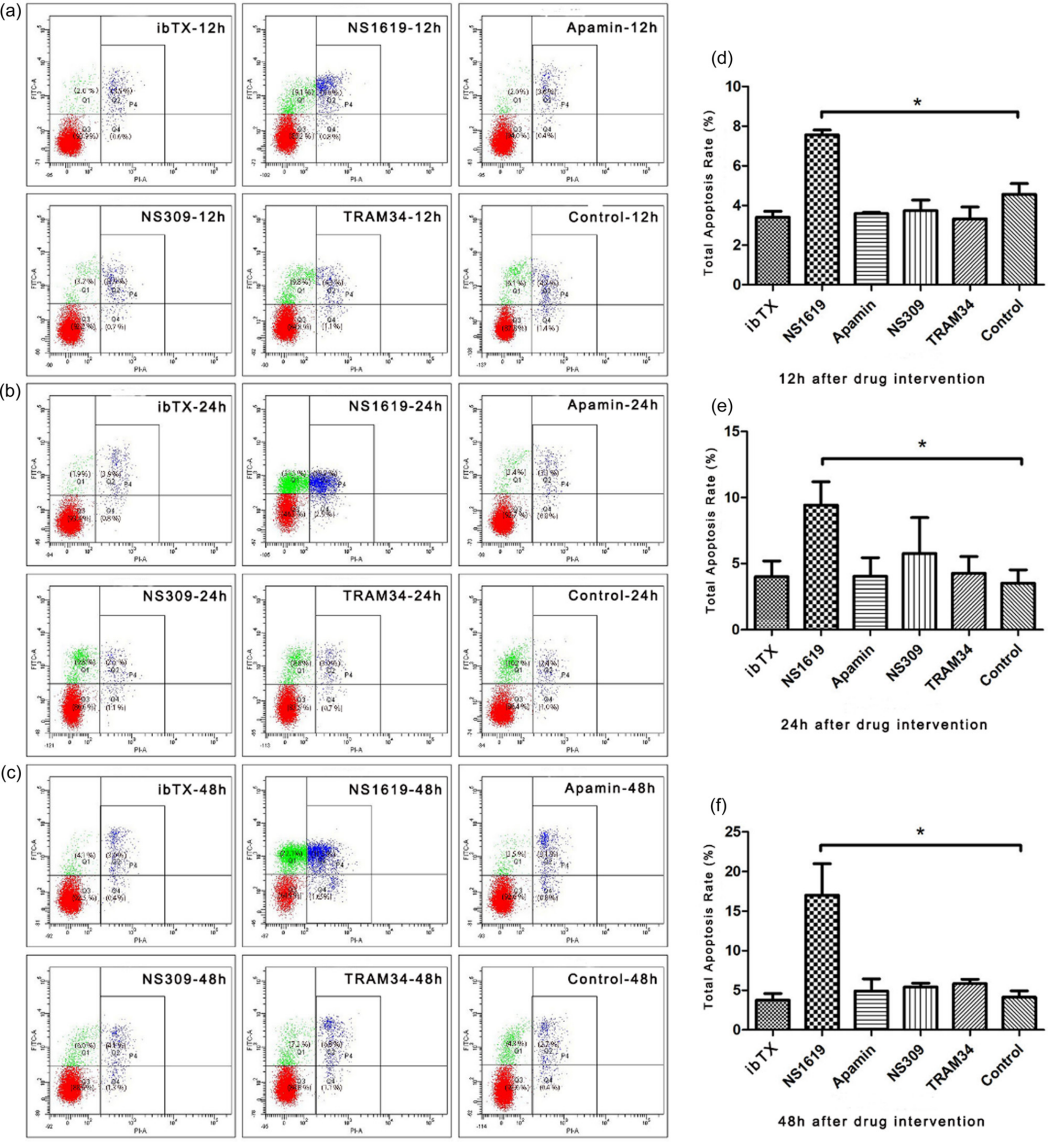
Apoptosis of HTR-8/SVneo cells after intervention on KCa channels. NS1619, NS309, ibTX, TRAM34, and Apamin were used to treat HTR-8/SVneo cells for 12, 24, and 48 h, respectively. Flow cytometry was used to detect the apoptosis rate of each group (a–c), and the total apoptosis rate was calculated and analyzed (d–f). Data are displayed as mean ± SD (*n* = 3). *P < 0.05.

Compared with the control group, the apoptosis rate of the NS1619 group after 12 h (7.57 ± 0.40%), 24 h (9.43 ± 3.06%), and 48 h (17.0 ± 3.98%) was significantly increased (*P* < 0.05) (as shown in Figure 5). However, no significant difference in apoptosis was observed between the other groups with drug intervention (such as ibTX, NS309, TRAM34, and Apamin) and the control group (*P* > 0.05). These results indicated that activating BKCa channels could promote the apoptosis of HTR-8/SVneo cells, while either activating IKCa and SKCa channels or inhibiting BKCa, IKCa, and SKCa channels did not affect the apoptosis of HTR-8/SVneo cells.

### 3.5 Migration of HTR-8/SVneo cells after intervention on KCa channels

**Figure 6:**
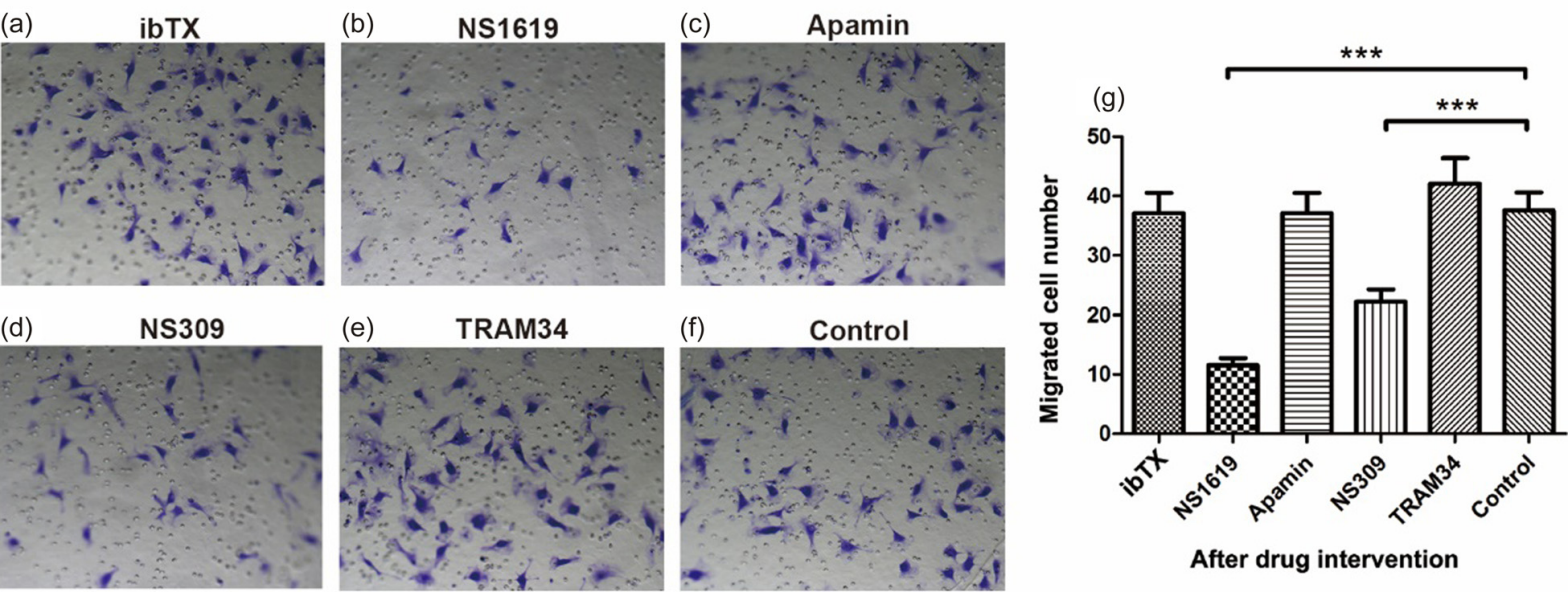
Migration of HTR-8/SVneo cells after intervention on KCa channels. (a–f) The average number of HTR-8/SVneo cells that passed through the transwell membrane after using ibTX, NS1619, Apamin, NS309, and TRAM34 under the microscope. (g) The number of cells passing through the membrane in each drug intervention group, compared with the control group. Data are displayed as mean ± SD (*n* = 3). ***P < 0.001.

The number of HTR-8/SVneo cells that passed through the transwell membrane decreased significantly in the channel activator groups (NS1619 and NS309) compared with the control group (Figure 6) (*P* < 0.001). However, there was no significant difference between the KCa channel-inhibiting group (ibTX, TRAM34, and Apamin) and the control group (*P* > 0.05). It indicated that activating BKCa, IKCa, and SKCa channels inhibited migration of HTR-8/SVneo cells, while inhibiting KCa channels did not exhibit the opposite effect.

## 4 Discussion

This research showed that the three subtypes of KCa channel were expressed in both human chorionic villi and HTR-8/SVneo cells. Either activating or inhibiting different KCa channels contributed to behavioral changes in trophoblast cell proliferation, apoptosis, and migration. It implied the significant role of KCa channels in the physiological functions of trophoblasts.

### 4.1 Localization and expression of KCa channels in human chorionic villi and trophoblast cell line

The expression of KCa channels has been found in female reproductive systems, such as the uterus [19], ovary [20], and placenta [12]. However, few studies evaluated the effect of KCa channels on the biological functions of trophoblast cells. In this study, three types of KCa channels were found to express in first-trimester chorionic villi and EVT cell line, where they were found mainly localized on the membrane and in the cytoplasm. These results suggested that KCa channels might play an important role in placental development. Some researchers confirmed that different intracellular localizations of KCa channels were associated with discrepant cellular functions in non-placental cells (e.g., intracellular trafficking [21] and in mitochondria [22]). Diaz et al. found that IKCa channels were localized in the nucleus and cytoplasm and on the membrane of CTBs that were isolated from term placentas during various differentiation stages. In addition, activating IKCa channels could markedly reduce CTB syncytialization [23]. However, studies on BKCa and SKCa channels in trophoblast cell functioning have not been reported. In this research, the expression of the BKCa and SKCa in trophoblast cells was substantiated; however, their roles in trophoblast syncytialization and their expression dynamics throughout gestation warrant further exploration.

### 4.2 Effects of KCa channel openers and inhibitors on trophoblast proliferation

KCa channels have been reported to play an important role in regulating cell proliferation and apoptosis in various tissue cells and cancer cells. A recent study showed that the overexpression of KCNN4 (potassium calcium-activated channel subfamily *N* member 4)-gene, which encodes IKCa channels, could markedly boost cell proliferation and increase the salutary effects of human heart explant-derived cells on cardiac function [13]. Activating IKCa channels could also promote tumor cell proliferation [24,25]. Overexpression of BKCa channels could enhance the proliferation and migration of endometrial cancer HEC-1-B cells [26]. Mound et al. found that activation of BKCa channels by type 3 of inositol 1,4,5-trisphosphate receptor could promote the proliferation stimulation of breast tumor cells [27]. However, SKCa channel activation led to the inhibition of proliferation and induction of differentiation in embryonic stem cells via Ras–Mek–Erk signaling cascade [28]. In this research, we found that the proliferation of EVTs was inhibited by BKCa, IKCa, and SKCa channel activation. The above findings implied a cell type-specific role of KCa channels in cell proliferation. Molecular mechanisms underlying these phenomena remain to be further explored.

### 4.3 Effects of KCa channel openers and inhibitors on trophoblast apoptosis

The three types of KCa channels seem to play different roles in apoptosis in different cell types. Zhu et al. found that BKCa channel activator NS1619 promoted apoptosis of vascular smooth muscle cells [10]. Chang et al. found that activation of BKCa channels significantly induced apoptosis but suppressed the proliferation of human embryonic kidney 293 cells under hyperglycemic conditions [29]. Consistent with Chang et al., our data suggested that NS1619 promoted apoptosis but suppressed the proliferation of trophoblast cells. Considering the importance of the balance between trophoblast proliferation and apoptosis in the event of placentation and recasting of uterine vessels [5], we speculated that BKCa channels might be involved in spiral artery remodeling during early gestation by mediating the dynamic balance between proliferation and apoptosis of trophoblasts.

Previous studies have reported that IKCa channel inhibitor TRAM34 had no significant effect on apoptosis of hepatocellular carcinoma [30]. In our study, we also did not observe the cytotoxicity of IKCa channels on trophoblast cells. It indicated that IKCa channels might affect the biological behavior of trophoblasts other than apoptosis, such as proliferation and migration.

Inhibition of SKCa channels appeared to be cytotoxic in various cell lines. Abdulkareem et al. found that the knockdown of SKCa channels in five widely studied breast cancer cell lines was accompanied by a decrease in Bcl-2 expression and an increase in both caspase-7 and caspase-9 expression, indicating that apoptosis was promoted [31]. Recently, our team also showed that the blockade of SKCa channels with apamin significantly promoted apoptosis in human umbilical vein endothelial cells [32]. However, a similar cytotoxic effect on HTR-8/SVneo cells was not observed, neither activating nor inhibiting SKCa channels, indicating that SKCa channels might not be involved in the regulation of trophoblast cell apoptosis.

### 4.4 Effects of KCa channel openers and inhibitors on trophoblast migration

The promotion of cell migration by KCa channels occurs in many cell types. Data from recent studies suggested that inhibition of BKCa channels could suppress the migration of rat mesangial cells [33], human endometrial adenocarcinoma cell line (Ishika cells) [34], and endometrial cancer HEC-1-B cells [26]. Similar effects of IKCa channels in cell migration were observed. It was reported that IKCa channels contributed to colorectal cancer cell migration and invasion by modulating both Ca^2+^ entry and ROS level [35]. SKCa knockdown suppressed the migration of MDA-MB-435s, a human breast cancer cell line; transient expression of SKCa channel protein in an SKCa-deficient cell line promoted cell migration [36]. Contrary to the above studies, our results found that activating BKCa, IKCa, and SKCa channels inhibited the migration of HTR-8/SVneo cells, suggesting that the roles of KCa channels in regulating EVTs migration might be different from that of cancer cells. Unfortunately, according to our previous studies, only a certain concentration of each KCa channel opener or inhibitor was used in this study [12,14,32]. Further studies about the effects of other different concentrations on cell biological effects will be conducted, which would help us better understand the biological roles of KCa channels in trophoblasts and placentation. In light that the migration and invasion of EVTs is fundamental to placentation [37], it could be inferred that KCa channels might be implicated in placental development and/or gestational pathologies.

To recapitulate, the role of KCa channels in placental development and placenta-derived diseases remains unknown. In this research, we proved that KCa channels were located in chorionic villi during early gestation and had participated in the proliferation, apoptosis, and migration of EVTs. Whether dysfunction of KCa channels is involved in the pathogenesis of pregnancy-related complications, such as recurrent miscarriage, preterm birth, PE, and FGR, will be addressed in our future research.
